# Is It Possible to Predict Pulmonary Complications and Mortality in Hematopoietic Stem Cell Transplantation Recipients from Pre-Transplantation Exhaled Nitric Oxide Levels?

**DOI:** 10.4274/tjh.2014.0159

**Published:** 2016-02-17

**Authors:** Nurdan Köktürk, Fatma Yıldırım, Müge Aydoğdu, Şahika Zeynep Akı, Zeynep Arzu Yeğin, Zübeyde Nur Özkurt, Elif Suyanı, İpek Kıvılcım Oğuzülgen, Gülsan Türköz Sucak

**Affiliations:** 1 Gazi University Faculty of Medicine, Department of Pulmonary Medicine, Ankara, Turkey; 2 Gazi University Faculty of Medicine, Department of Hematology, Ankara, Turkey

**Keywords:** Hematopoietic stem cell transplantation, Exhaled nitric oxide, Pulmonary complications, mortality

## Abstract

**Objective:**

Chemo/radiotherapy-induced free oxygen radicals and reactive oxygen derivatives contribute to the development of early and late transplantation-related pulmonary and extra-pulmonary complications in hematopoietic stem cell transplantation (HSCT) recipients. It has been proposed that an increase in fractional exhaled nitric oxide (FeNO) level indicates oxidative stress and inflammation in the airways. The aim of this prospective study is to evaluate the pre-transplantation FeNO levels in HSCT patients and to search for its role in predicting post-transplantation pulmonary complications and mortality.

**Materials and Methods:**

HSCT patients were included in the study prospectively between October 2009 and July 2011. Pre-transplantation FeNO levels were measured with a NIOX MINO® device prior to conditioning regimens. All patients were monitored prospectively for post-transplantation pulmonary complications with medical history, physical examination, chest X-ray, and pulmonary function tests.

**Results:**

A total of 56 patients (33 autologous, 23 allogeneic) with mean age of 45±13 years were included in the study, among whom 40 (71%) were male. Pre-transplantation FeNO level of the whole study group was found to be 24±13 (mean ± standard deviation) parts per billion (ppb). The FeNO level in allogeneic HSCT recipients was 19±6 ppb while it was 27±15 ppb in autologous HSCT recipients (p=0.042). No significant correlation was found between the pre-transplantation chemotherapy and radiotherapy protocols and baseline FeNO levels (p>0.05). Post-transplantation pulmonary toxicity was identified in 12 (21%) patients and no significant relationship was found between baseline FeNO levels and pulmonary toxicity. The survival rate of the whole study group for 1 year after transplantation was 70%. No significant relationship was identified between baseline FeNO values and survival (FeNO 19±7 ppb in patients who died and 26±15 ppb in the survivors; p=0.114).

**Conclusion:**

Pre-transplantation FeNO measurement does not seem to have a role in predicting post-transplantation pulmonary complications and mortality.

## Abstract (tr)

**Amaç:**

Hematopoetik kök hücre nakli (HKHN) hastalarında, nakil öncesi kemoterapi/radyoterapi etkisi ile oluşan serbest oksijen radikalleri ve reaktif oksijen türevleri pek çok nakil ilişkili erken ve geç pulmoner ve nonpulmoner komplikasyonun oluşmasında rol oynamaktadır. Ekshale nitrik oksit (NO) düzeyindeki artışın hava yollarındaki oksidatif stresi ve enflamasyonu yansıttığı ileri sürülmektedir. Bu prospektif çalışmada amacımız HKHN hastalarında nakil öncesi bakılan ekshale NO düzeylerinin nakil sonrası gelişen pulmoner komplikasyonlar ve sağkalım üzerine etkisinin araştırılması idi.

**Gereç ve Yöntemler:**

Ekim 2009-Temmuz 2011 tarihleri arasında HKHN uygulanan hastalar prospektif olarak çalışmaya alındılar. Nakil öncesi ekshalasyon havasında NO ölçümleri NIOX MINO® cihazı ile yapıldı. Bütün hastalar transplantasyon sonrası prospektif olarak anemnez, fizik muayene, akciğer filmi ve solunum fonksiyon testleri ile pulmoner komplikasyon açısından takip edildi.

**Bulgular:**

Çalışmaya yaş ortalamaları 45±13 yıl olan 40’ı (71%) erkek toplam 56 hasta (33 otolog, 23 allojeneik) dahil edildi. Nakil öncesi ekshale NO düzeyi tüm grupta 24±13 ppb (ortalama ± standart sapma) (median: 22; minimum-maksimum: 5-75) bulundu. Allojeneik HKHN uygulanan hastalarda ekshale NO düzeyi 19±6 ppm; otolog nakil uygulananlarda 27±15 ppm olarak bulundu (p=0,042). Nakil öncesi uygulanan kemoterapi ve radyoterapi rejimleri ile bazal NO düzeyleri arasında anlamlı korelasyon bulunmadı (p>0,05). Nakil sonrası pulmoner toksisite 12 (%21) hastada saptandı. Bazal NO değeri ile pulmoner toksisite arasında anlamlı ilişki saptanmadı. Tüm çalışma grubunda sağkalım oranı %70 olarak bulundu. Nakil öncesi ekshale NO düzeyinin sağ kalım üzerinde etkisinin olmadığı görüldü (eksitus grubunda 19±7 ppm, taburcu grubunda 26±15 ppm, p=0,114).

**Sonuç:**

HKHN hastalarında nakil öncesi ekshale NO ölçümü nakil sonrası pulmoner komplikasyon gelişimini ve mortaliteyi öngörmede kullanılabilir gözükmemektedir.

## INTRODUCTION

Hematopoietic stem cell transplantation (HSCT) is an important treatment option for several malignant and non-malignant hematological diseases. However, pulmonary complications such as idiopathic pulmonary syndromes, bronchiolitis obliterans organizing pneumonia (BOOP), and infections and graft-versus-host disease (GVHD) developing after bone marrow transplantation have a negative impact on outcome. Chemo/radiotherapy-induced oxidative stress occurring prior to HSCT is claimed to contribute to development of many early and late transplantation-related pulmonary complications [1,2,3,4,5]. A marker of bronchial inflammation might guide in predicting HSCT-related pulmonary pathology.

Nitric oxide (NO) is an endogenous regulator molecule that is synthesized in the body from L-arginine by the enzyme NO synthase. NO in the airways is measured after reaction with ozone by chemiluminescence method. Fractional exhaled NO (FeNO) has been shown to increase as a non-invasive marker of inflammation especially in bronchiectasis, bronchial asthma, tuberculosis, acute exacerbation of chronic obstructive pulmonary disease (COPD), and many other systemic and autoimmune diseases such as systemic lupus erythematosus, systemic sclerosis, and cirrhosis of the liver. Its level increases in parallel with the increase in the level of inflammation and tends to decline in a short period of time after anti-inflammatory therapies [6,7,8].

Our group demonstrated a significant relationship between pre-transplantation diffusion capacity of the lungs for carbon monoxide adjusted for hemoglobin (DLCO adj) levels and development of post-transplantation sinusoidal obstruction syndrome after transplantation in a previous study [9]. We hypothesized whether exhaled FeNO, a simple and non-invasive measurement that has been shown to be increased in many inflammatory conditions, might show pre-transplantation inflammation and endothelial injury and could possibly predict post-transplantation pulmonary complications and mortality.

## MATERIALS AND METHODS

### Study Subjects

After receiving the approval of our institutional review board, 56 patients were enrolled in the study prospectively between October 2009 and July 2011. Inclusion criteria were age above 18 years, being a candidate for allogeneic or autologous stem cell transplantation, and signing an informed consent form to participate in the study and for the use of their medical records. Patients who were younger than 18 years of age; who smoked through the last 6 months; who had asthma, COPD, bronchial hyperreactivity, or upper or lower respiratory tract infection in the last 4 weeks; who were diagnosed to have had activation of the underlying disease in the last 4 weeks; and who used L-arginine, phosphodiesterase inhibitors, or nitrate were excluded from the study.

### Graft-Versus-Host Disease

Cyclosporine and methotrexate was the standard prophylaxis regimen for GVHD. The assessment and grading of acute and chronic GVHD was primarily based on clinical findings and pulmonary function test (PFT) results. Overall grade of acute GVHD and severity of organ involvement was assessed on a 0-to-4 scale according to the original Seattle criteria. Acute GVHD was considered present if a grade of at least 2 was assigned. Pulmonary toxicities were also graded on a 0-to-4 scale according to these criteria. Chronic GVHD was defined as GVHD occurring 100 days or more after HSCT. GVHD was treated with 1-2 mg/kg/day of prednisolone.

### Antimicrobial Prophylaxis

Antimicrobial prophylaxis, which was performed with acyclovir and fluconazole, was given from the beginning of the conditioning regimen until day +180 post-transplantation for autologous and until discontinuation of the immunosuppressive therapy for allogenic HSCT recipients. All patients received trimethoprim-sulfamethoxazole orally as prophylaxis against Pneumocystis jirovecii beginning from the conditioning regimen until 1 day before stem cell infusion and from neutrophil engraftment to 6 months post-transplantation for autologous and until the discontinuation of the immunosuppressive therapy for allogenic HSCT.

### Fractional Exhaled Nitric Oxide Measurements

FeNO levels were measured with a NIOX MINO® device prior to HSCT. Each subject inhaled NO-free air to the total lung capacity (TLC) and expired as long as possible at 4 different flow rates (50, 100, 150, and 200 mL/s) against a resistance of 10 cm H2O/L/s as previously described. The respiratory tract contains both forms of nitric oxide synthetase enzymes, inducible and endothelial. The FeNO measurement represents the total NO of the respiratory tract [10].

### Pulmonary Follow-Up

PFTs were routinely performed for detection of underlying ventilatory abnormalities and for assessment of baseline lung function. The following parameters were measured: forced expiratory volume in the first second (FEV1), forced vital capacity (FVC), FEV1/FVC ratio, and TLC. In order to determine non-infectious pulmonary complications, serial PFTs of patients were also performed at 3, 6, 9, and 12 months after HSCT. DLCO adj, DLCO per unit alveolar volume (DLCO VA), and DLCO adjusted for alveolar volume (DLCO VA/adj) were also measured.

### Pulmonary Toxicity Definitions

Infectious pulmonary complications were defined as pulmonary infections with clinical signs of fever, dyspnea, and crackles, proven by radiologic infiltrates on chest X-ray and in microbiological samples. Microbiological samples were obtained from the data of microbiological analyses, including direct microscopy or culture of sputum or bronchoalveolar lavage fluid.

Non-infectious pulmonary complications were defined as the new onset of an obstructive pulmonary defect clinically manifested by dyspnea on exertion, cough, or wheezing. Evidence of obstructive defect is revealed in PFTs. Non-infectious pulmonary complication was accepted as bronchiolitis obliterans syndrome (BOS), because BOS is a clinical term defined by pulmonary function changes rather than histology. Patients were classified as having BOS if they showed FVC % predicted of >80% and FEV1/FVC of <70% [11,12].

### Statistical Analysis

Statistics were calculated using SPSS 15.0 for Windows. Continuous variables are presented as mean±SD and categorical variables as percentages. Patients with and without pulmonary complications were compared using the chi-square test for categorical variables and the t-test and Mann-Whitney U test for continuous variables. Pearson and Spearman correlation tests were used to determine the relationship between basal FeNO levels and pulmonary complications. Values of p<0.05 were considered statistically significant.

The allogeneic and autologous HSCT groups were compared according to their basal FeNO levels as high and low FeNO groups, but there was no cut-off point. Therefore, we divided the groups according to their median basal FeNO group. Pearson and Spearman correlation tests were used to determine the relationships between groups.

Kaplan-Meier survival analysis was performed for comparing the progression-free survival (PFS) and overall survival among allogenic and autologous patients with low and high basal FeNO levels. A difference was considered statistically significant when p<0.05 by log-rank.

## RESULTS

### Patient Characteristics

Fifty-six patients were included in the study; 33 received autologous and 23 received allogeneic stem cells. Forty (71%) patients were male, and the mean age of the patients was 45±13 years. The median age of the autologous HSCT group was higher than that of the allogenic HSCT group (53 vs. 34, p=0.001). The basal FeNO level of the autologous HSCT group was higher than that of the allogenic HSCT group (26.8±15.4 vs. 18.9±6.2 ppb, p=0.042) (Tables 1 and 2).

In the allogeneic and autologous HSCT groups there were no significant relations with regard to age, sex, diagnosis, exitus rate, progression rate, and total and infectious pulmonary complications for low and high basal FeNO levels (p>0.05) (Table 3).

Basal PFT values of the groups with pulmonary complications and without pulmonary complications were similar (Table 4).

### Infectious Pulmonary Toxicities

In the allogeneic HSCT group, 3 of the infections were bacterial, 2 were fungal, and 1 was viral. In the autologous HSCT group, the infectious agent was bacterial in one patient and Pneumocystis jirovecii in the other patient. All of the pulmonary infections were with febrile neutropenia and pneumonia. The incidence of invasive pulmonary aspergillosis was 3.7%.

### Non-Infectious Pulmonary Toxicities

In the allogeneic HSCT group, BOS was diagnosed in 2 (9%) patients, and in the autologous HSCT group BOS was detected in 2 (6%) patients.

In the autologous HSCT group, one patient had acute GVHD. This patient was a woman and had 2 children. This is a rare complication and it was thought to be the result of maternal antigen activation [13,14].

### Survival

There were no deaths in the allogeneic and autologous groups within the first 100 days after HSCT. The survival rate of the whole study group for 1 year after transplantation was 70%.

The mean value of the pre-transplantation FeNO was 19±7 ppb in the exitus group, while it was 26±15 ppb in the survivors in the total study group. There was no significant relationship between baseline FeNO and survival (p=0.114).

The median follow-up of 33 patients with autologous HSCT was 424 days (154-766 days). Survival rate of these patients at the end of the follow-up period was 78.8%. Six patients succumbed to their underlying disease after autologous HSCT, whereas 1 patient died due to transplant-related causes. None of patients died due to only pulmonary complications. Pre-transplantation basal FeNO levels did not have an impact on survival (p>0.05). After autologous HSCT, 4 of the 33 patients (12.1%) developed grade 3-4 pulmonary toxicity. Basal FeNO levels had no impact on pulmonary toxicity (p>0.05).

The median follow-up of the 23 allogeneic HSCT recipients was 203 days (10-774 days). The survival rate of these patients at the end of the follow-up period was 56.5%. While 6 patients died due to transplant-related causes, 4 patients died of disease-related causes. Pre-transplantation FeNO level was 19±6 ppb in allogeneic HSCT recipients. Pre-transplantation basal FeNO levels had no impact on survival, as well (p>0.05). Seven of the 23 patients (30.4%) developed grade 3-4 pulmonary toxicity after allogeneic HSCT. One patient had grade 1-2 toxicities and this patient was excluded from analysis. No relationship was found between the basal FeNO levels and the development of pulmonary toxicity (p>0.05).,

Mean PFS was 246.3±210.1 days in the allogeneic HSCT group; for autologous HSCT patients, mean PFS was 366.7±199.1. There were no significant associations between PFS and basal FeNO levels of low and high basal FeNO group patients in either the allogeneic (p=0.460) or the autologous (p=0.52) HSCT group when examined with Kaplan-Meier analysis and log-rank test.

## DISCUSSION

We hypothesized that pre-transplantation FeNO could be a surrogate biomarker demonstrating pre-existing pulmonary inflammation and/or pre-transplantation injury caused by the oxidative stress due to previous chemotherapies, radiation, and infections. We therefore measured pre-transplantation FeNO levels and investigated whether they had an impact on post-transplantation pulmonary toxicity and mortality. However, our results failed to demonstrate an impact of FeNO levels on transplantation outcomes. There was neither a correlation between baseline FeNO levels and pre-transplantation induction chemotherapy and radiotherapy protocols nor post-transplantation pulmonary complications and mortality.

Pulmonary complications are major causes of morbidity and mortality after HSCT. Unfortunately, there is currently no established marker that is non-invasive and can predict the pulmonary complications and guide preventive strategies or risk-stratified transplant techniques. A significant correlation has been observed between the levels of serum nitrite/nitrate and host-versus-graft and graft-versus host reactions in rats [15] and humans [4,5] in previous studies. Furthermore, measurement of NO in exhaled air has been proposed for the assessment of individual “pulmonary” risk status amongst various other factors in adults.

Haddad et al. [16] studied a mouse model of idiopathic pneumonia syndrome following bone marrow transplantation. They showed that alveolar macrophages, after being stimulated by allogeneic T cells of graft origin, express higher NO synthase levels and thus produce more NO. Cyclophosphamide in their model stimulated superoxide production by alveolar macrophages. They concluded that the resultant higher NO and superoxide levels might have led to production of peroxynitrites and nitrotyrosines, which mediated lung damage.

Several investigators assessed the usefulness of FeNO for the early detection of chronic GVHD in HSCT recipients. Kanamori et al. [17] published a case series of adults with BOOP after HSCT with FeNO levels above 36 ppb, suggesting that elevated FeNO may be indicative of pulmonary complications after HSCT. Increased FeNO production in their cases also suggests that bone marrow transplantation-related BOOP might be a manifestation of chronic GVHD. They proposed that the FeNO measurement was useful in monitoring inflammatory complications after HSCT.

Two previous reports studied the concentration of exhaled NO in patients after lung transplantation [8,18]. Neurohr et al. studied 166 consecutive lung transplantation recipients [18]. Those patients received no induction therapy and were maintained with standard care on triple immunosuppression with corticosteroids, tacrolimus, and mycophenolate mofetil. A total of 611 FeNO measurements were classified depending on BOS stage at the time of assessment and course during a minimum follow-up of 3 months: stable non-BOS, unstable non-BOS, stable BOS, and unstable BOS. FeNO was significantly increased prior to the unstable course in comparison to the stable counterparts (non-BOS: 28.9±1.2 ppb, 16.4±0.8 ppb, and BOS: 32.5±1.3 ppb, 15.3±0.8 ppb, respectively). Their report demonstrated that elevated levels of FeNO constituted an increased risk for future BOS and preceded further deterioration in transplant recipients.

Another report studying FeNO in patients after autologous HSCT is that of Qureshi et al. [19]. In their study, FeNO was significantly increased following autologous peripheral HSCT and correlated with reduction in DLCO. Mean FeNO increased from 12.54±1.32 ppb before HSCT to 21.26±1.94 ppb at 6 weeks and 25.28±3.31 ppb at 24 weeks. The exhaled FeNO was determined before and after the conditioning regimen and showed a significant and progressive increase after the conditioning, suggesting chemotherapy-related pulmonary toxicity. FeNO was also measured prior to the conditioning regimen in the current study in order to determine whether it could be used as a biomarker that defines pre-transplantation pulmonary risk status. However, FeNO unfortunately failed to demonstrate such a predictive value. Fazekas et al. [20] evaluated the correlation of FeNO and pulmonary complications in 30 pediatric HSCT patients. They measured FeNO 10 days before HSCT and at day 0, day 28, and day 60 of HSCT. Similar to our results pre-transplantation, FeNO levels were not different in patients with and without post-transplantation pulmonary complications. However, children with any kind of pulmonary complications until day 100 of HSCT had higher FeNO levels at day 0 than children without early respiratory pathology, suggesting the role of conditioning chemotherapy rather than the induction regimens prior to transplantation.

In previous studies, age was noted as a predictor of FeNO in children [21,22]. However, the influence of age is controversial in healthy adults [23,24,25]. In our study, the autologous HSCT group was older than the allogenic HSCT group, and the basal FeNO level of the autologous HSCT group was higher than that of allogenic HSCT group. We attributed this difference to advanced age.

The limitation of our study is the lack of consecutive FeNO measurements within the long-term follow-up. By serial measurement of FeNO, its potential as a non-invasive marker for continuous risk stratification of HSCT patients for determining pulmonary complications might be better identified.

## CONCLUSION

We conclude that pre-transplantation FeNO levels do not seem to be of value as a marker of post-transplantation pulmonary complications and mortality pre-transplantation. Further studies are required to designate a pre-transplantation surrogate marker of post-transplantation pulmonary toxicity.

## Ethics

Ethics Committee Approval: Gazi University Ethics Committee (Approval number: 43/10.01.2009), Informed Consent: It was taken.

## Figures and Tables

**Table 1:**
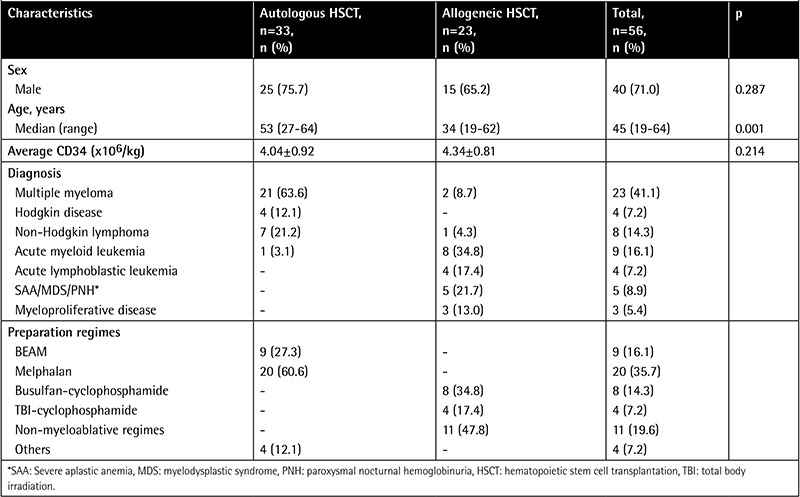
Baseline clinical characteristics of patients undergoing hematopoietic stem cell transplantation.

**Table 2:**
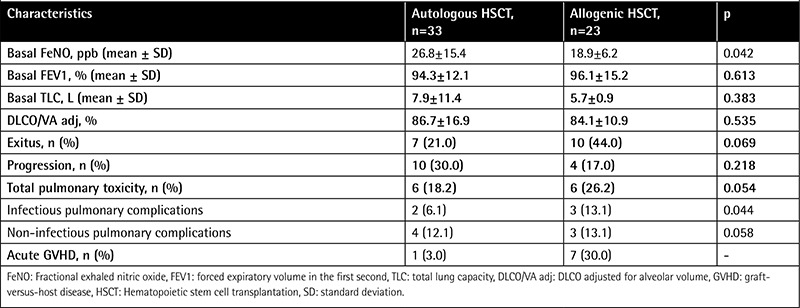
Fractional exhaled nitric oxide levels, pulmonary function test measurements, and pulmonary complications.

**Table 3:**
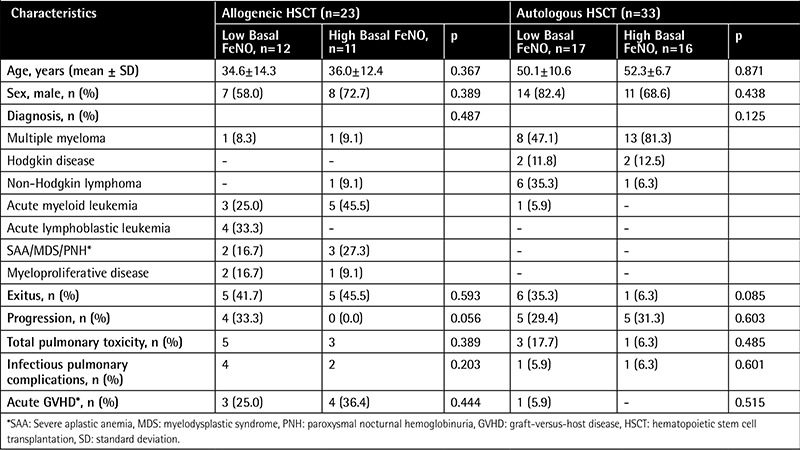
Comparison of allogeneic and autologous hematopoietic stem cell transplantation groups according to basal fractional exhaled nitric oxide.

**Table 4:**
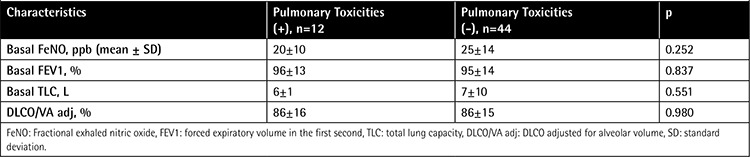
Characteristics of patients with and without pulmonary toxicities.
